# Inhalation Injury Grading Using Transfer Learning Based on Bronchoscopy Images and Mechanical Ventilation Period

**DOI:** 10.3390/s22239430

**Published:** 2022-12-02

**Authors:** Yifan Li, Alan W. Pang, Jad Zeitouni, Ferris Zeitouni, Kirby Mateja, John A. Griswold, Jo Woon Chong

**Affiliations:** 1 Department of Electrical and Computer Engineering, Texas Tech University, Lubbock, TX 79409, USA; 2 Department of Surgery, Texas Tech University Health Sciences Center, Lubbock, TX 79430, USA; 3 School of Medicine, Texas Tech University Health Sciences Center, Lubbock, TX 79430, USA

**Keywords:** inhalation injury, deep learning, convolutional neural networks (cnn), transfer learning

## Abstract

The abbreviated injury score (AIS) is commonly used as a grading system for inhalation injuries. While inhalation injury grades have inconsistently been shown to correlate positively with the time mechanical ventilation is needed, grading is subjective and relies heavily on the clinicians’ experience and expertise. Additionally, no correlation has been shown between these patients’ inhalation injury grades and outcomes. In this paper, we propose a novel inhalation injury grading method which uses deep learning algorithms in bronchoscopy images to determine the injury grade from the carbonaceous deposits, blistering, and fibrin casts in the bronchoscopy images. The proposed method adopts transfer learning and data augmentation concepts to enhance the accuracy performance to avoid overfitting. We tested our proposed model on the bronchoscopy images acquired from eighteen patients who had suffered inhalation injuries, with the degree of severity 1, 2, 3, 4, 5, or 6. As performance metrics, we consider accuracy, sensitivity, specificity, F-1 score, and precision. Experimental results show that our proposed method, with both transfer learning and data augmentation components, provides an overall 86.11% accuracy. Moreover, the experimental results also show that the performance of the proposed method outperforms the method without transfer learning or data augmentation.

## 1. Introduction

**Figure 1:**
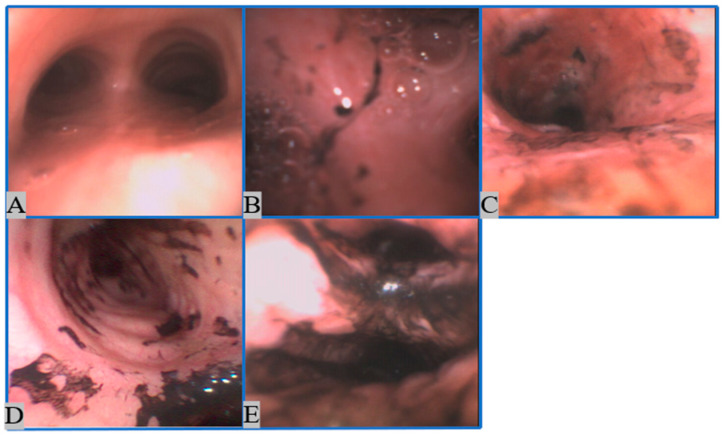
An example of grading of inhalation injury using the abbreviated injury score (AIS). (**A**)—no injury; (**B**)—mild injury; (**C**)—moderate injury; (**D**)—severe injury; (**E**)—massive injury.

A smoke inhalation injury is one of the significant complications of burn injuries [1]. Among the reported burn injuries in the United States (US) in 2022, 7.7% included smoke inhalation injuries [1]. Burn patients with an inhalation injury exhibit higher mortality rates than those without inhalation injuries [2]. For example, from 2009 to 2018, the mortality rate of burn patients without inhalation injuries was 2.9%, while in the presence of smoke inhalation injuries, the mortality rate increased by 20%. Specifically, the mortality increased by up to 60% with concurrent pneumonia [2]. In addition, smoke inhalation also increases the length of hospital stay [3,4]. The pathophysiology of inhalation injury is caused by the following three reasons: (1) direct thermal injury to the upper airway, (2) chemical irritation to the lower subglottic airway by smoke, and (3) metabolic injury from specific chemicals from the smoke, such as hydrogen cyanide [5]. Airway inflammation and pulmonary shunting after the inhalation injury both lead to hypoxemia [6]. Moreover, inhalation injuries may impair macrophage function, leading to impaired ciliary clearance [7,8]. Inhibition of alveolar macrophages may expose patients to bacterial infections and predispose them to developing pneumonia (a leading cause of death in burn injury) [9]. Furthermore, patients with cutaneous injuries are at additional risk of developing pneumonia [9]. Currently, the most widely used grading system for inhalation injury is the abbreviated injury score (AIS) [10], which categorizes the severity of the injury on a scale of (1) grade 0 (no injury), (2) grade 1 (mild injury), (3) grade 2 (moderate injury), (4) grade 3 (severe injury), and (5) grade 4 (massive injury). Examples of bronchoscopy imaging acquired from Grades 0–4 patients are shown in Figure 1 [11]. However, studies have shown an inconsistent cause-and-effect relationship between inhalation injury grade and the period during which the patient requires mechanical ventilation [12]. Additionally, the inhalation injury grade from the current AIS is not highly correlated with mortality [10,11,12,13], either.

The currently widely employed inhalation injury grading systems, such as AIS, are based on the findings of the initial bronchoscopy examination. The lack of consensus on diagnosis, grading, and prognosis of inhalation injury stems from the limitations of the AIS grading system. For example, since bronchoscopy is not easy to identify, narrow distal airway changes, diagnosis and grading likely depend on the image quality and interpretation [14]. Additionally, the subjectivity of this grading system places reliance on the examiner’s expertise for accuracy [14].

Deep learning and artificial intelligence have shown promising medical imaging classification capabilities [15]. For example, a convolutional neural network (CNN), a type of artificial neural network, is widely used for medical image classification and feature recognition [16]. Our proposed inhalation injury grading method is based on machine learning and artificial intelligence and aims to address the limitations of the currently utilized method, such as AIS. As a result, the proposed method allows consistency and objectivity in diagnosis. To the best of our knowledge, the application of deep learning artificial intelligence in grading the severity of inhalation injuries has not been done. 

### 1.1. Related Work

The diagnosis of inhalation injury currently relies on clinical exam findings and bronchoscopy evidence. The development of grading systems and the use of modalities such as chest computed tomography allow for a more detailed assessment of inhalation injury and enhance predictive power [17]. Currently, to the best of our knowledge, there is no literature discussing the application of deep learning models to diagnose the severity of inhalational injuries or the development of inhalation injury grading systems. 

Machine learning has been applied in burn care recently. Similar to the treatment of inhalational injury, the diagnosis and staging of burn injuries is also largely subjective and heavily reliant on clinician experience. Artificial intelligence and automated machine learning models are currently being proposed for the diagnosis and staging of burn wounds as they would provide clinicians with an automatic and reliable diagnostic tool. Yadav et al. developed a burn classification model using a support vector machine to diagnose burn injuries [18]. This model aimed to classify burn injuries into two categories, ones that needed grafting and ones that did not. Using burn injury skin images from the biomedical image processing database, their model showed an accuracy of 82.43% [18]. Rangel-Olvera et al. proposed a burn detection method based on using a sparse representation of feature vectors with over-redundant dictionaries, which provides 95.65% sensitivity and 94.02% precision [19]. Suha et al. applied CNN [20], and Lee et al. applied encoder–decoder CNN [21] to burn images to estimate burn severity. In [20], the CNN with transfer learning was trained to classify images according to their burn severity as a first-, second-, or third-degree burn, and the method provided 95.63% accuracy. In [21], encoder–decoder CNN classified burn depth based on altered tissue morphology and yielded 99% accuracy, 98% sensitivity, and 100% specificity. Moreover, a deep convolutional neural network-based body part-specific burns severity assessment model (BPBSAM) was investigated by Chauhan et al. [22] to predict the severity of a burn by applying body part-specific SVM. This is trained with CNN features which are extracted from the body part image of the burn. This model showed 84.85% accuracy on the BI test dataset, which included a blood test and ultrasound examination [22]. 

Optical coherence tomography (OCT) and Raman spectroscopy (RS) are two non-invasive optical modalities also used to extract textural and spectral wave features [23,24,25]. In [23], SVM and random forest algorithms are applied to OCT and RS, and 85% accuracy was achieved [23]. Spatial frequency domain imaging (SFDI) has also been used to characterize skin burns. An SVM can be trained with SFDI reflectance data at multiple spatial frequencies and can reach an accuracy of 92.5% [24]. These studies show that machine learning effectively diagnoses and manages burn care [25].

While inhalation injuries are a uniquely burn-related complication, imaging techniques on bronchoscopy images are applied in other fields, such as pulmonology [26,27]. Machine learning has been utilized and shown to accurately classify fibrotic lung disease using CT imaging, diagnose and differentiate COVID-19-associated pneumonia from other pneumonia, and even accurately diagnose lung cancer using low-dose CT imaging [26]. One study took this use of machine learning even further. Bronchoscopy imaging is used to visualize lung cancer, but physicians cannot diagnose the specific type of lung cancer. A recent study successfully used machine learning to estimate particular types of lung cancer using bronchoscopy imaging at an accuracy of 86% [27]. The diagnoses made were shown to be specific and accurate [27]. This is something we aimed to emulate in our study. We set out to utilize bronchoscopy imaging to accurately diagnose a specific grade of inhalation injury.

Transfer learning has proven to be effective in handling problems without large datasets, such as medical imaging [28]. Transfer learning retrains deep networks, pre-trained with a large amount of data, to solve the problem of data scarcity. VGG-19 [29], which is a form of CNN-based architecture, achieves an F1 score of 0.81–1 in COVID-19 recognition. In this experiment, a dataset was used which consisted of X-Ray images, Ultrasound images, and computerized tomography (CT) images [14]. Compared with AlexNet [30], GoogLeNet [31], and VGG-16 [32], ResNet-50 [33] achieves the highest accuracy of 56.09–81.41%, recognizing common thorax diseases on the ChestX-ray8 dataset, which contains chest X-ray images from 32,717 patients with eight common thorax diseases [34]. Moreover, a comprehensive multi-source dataset, built with X-ray and CT scan images for COVID-19 detection, proves that the AlexNet model can provide an accuracy of up to 98% [35]. ResNet-50 is also proven effective in recognizing malignant and benign tissues on CT scan images and achieves an accuracy of 99% [36]. For the cases where the learned features between the raw image and medical images mismatch, a novel transfer learning method has been shown to be promising in overcome the previous shortcomings by training deep learning models on a large unlabeled medical image dataset. This is then followed by the transfer of knowledge to train a deep learning model on the basis of a dataset comprised of a small number of labeled medical images [37]. The contrast-limited adaptive histogram equalization (CLAHE) method, which is used for enhancing the details, textures, and local contrast of the images, is improved with a log-normalization function that normalizes the intensity contrast of the images to normalized-CLAHE (N-CLAHE) [38]. Self-supervised learning (SSL), federated learning (FL), and generative adversarial network (GAN) methods are also proven to be applicable in the biomedical area [39]. Additionally, with transfer learning, ResNet 101 achieved the best accuracy of 95% in the skin burn diagnosis area [40].

### 1.2. Contribution

The main contributions of this paper are as follows:

- We propose a novel grading method for evaluating the severity of inhalation injury. Conventional inhalation diagnostic methods focused on the percentage of inhalation injury. However, our proposed inhalation diagnostic method is novel in that the method determines the severity of inhalation injuries based on our proposed deep machine learning algorithm with bronchoscopy images. Moreover, compared to the current manual grading system which depends on examiners, our proposed method gives quantitative and consistent results, which do not depend on inconsistent and subjective examiners’ decisions.
- Our proposed algorithm provides functionality that optimizes the hyperparameters of its deep machine learning model in terms of prediction accuracy of grading the severity of inhalation injuries. These include factors such as learning rate, drop period, max epochs, and mini-batch size. To achieve this, data augmentation and typical CNN-based models were also implemented for comparison with our proposed transfer learning method and exploration of higher performances. As a result, the proposed algorithm provides an average testing accuracy of 86.11%, which shows the potential to predict the severity of inhalation injuries.
- We analyze the impact of data augmentation and transfer learning by including or excluding these factors, respectively, in or from the algorithm. That is, we evaluate accuracy performance, in this paper, for the following combinations of methods, factors and paramteres: (1) transfer learning with data augmentation, (2) transfer learning without data augmentation, (3) non-transfer learning with data augmentation, and (4) non-transfer learning with data augmentation.

### 1.3. Paper Organization

The rest of this paper is organized as follows. In Section 2, we explain the process of developing the dataset and introduce the methods we applied in this study. In Section 3, we display the experiment results. We analyze the experiment results, compare our proposed method with previous ones, highlight the contribution of this study, and discuss the future steps we could undertake in Section 4.

## 2. Materials and Methods

### 2.1. Dataset Development

#### 2.1.1. Image Collection

The images which are used in this paper were collected by Drs. Griswold and Pang, both burn surgeons at the Timothy J. Harnar Regional Burn Center/Department of Surgery at Texas Tech University Health Science Center (TTUHSC) following the IRB (IRB#00000096) approved by the institutional review board for the protection of human subjects. Bronchoscopy is the gold standard used for evaluating airway and diagnosing inhalational injuries. A bronchoscopy, in this instance, is typically performed by the physician or physician trainees (residents). Learning to perform a bronchoscopy is included in medical training.

Additionally, it should be noted that there are few options to visualize an inhalation injury other than using a bronchoscopy. The diagnosis of the inhalational injury is made by the physician performing the bronchoscopy through the visualization of carbonaceous deposits, blistering, or fibrin casts in the bronchial tree. This diagnosis is entirely subjective and dependent on the physician. This is what primarily led us to develop a program utilizing machine learning and specific parameters to better diagnose and grade inhalation injuries. The way we visualize the injuries is no different from the current standard. The only change we make is by implementing an objective measurement (machine learning model) instead of the traditional subjective measurement form (physician determination and discretion). During the image collection, a thin, flexible, tubular camera (bronchoscope) is passed through the patient’s endotracheal tube and into the bronchi, where the bronchoscope takes images of injured bronchi.

**Figure 2:**
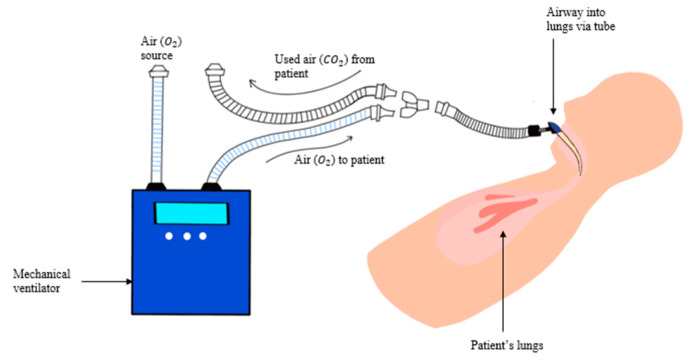
An example of a mechanical ventilator. The ventilator pulls air and extracts oxygen (O_2_) from an external source. The patient receives the oxygen from the tube passing into the lungs. The mechanical ventilator also removes CO_2_ from the patient’s lungs. The ventilator can be adjusted to control the rate and amount of ventilation.

A mechanical ventilator machine is an assistant tool that respiratory therapists and physicians use to treat respiratory failure patients, especially after sustaining an inhalational injury. It acts as a bellow to move air in and out of the lungs, as shown in Figure 2. Therapists and doctors set the ventilator to control how often it pushes air into the lungs and how much air is received.

The current grading systems for severity grading of inhalation injuries have an inconsistent cause-and-effect relationship between grades and the period when patients require mechanical ventilation. The current AIS grades are not highly correlated with mortality [11,12,13]. However, inhalation injury grades are proven to be highly associated with mortality, the period during which patients require mechanical ventilation and f fluid resuscitation [12].

**Figure 3:**
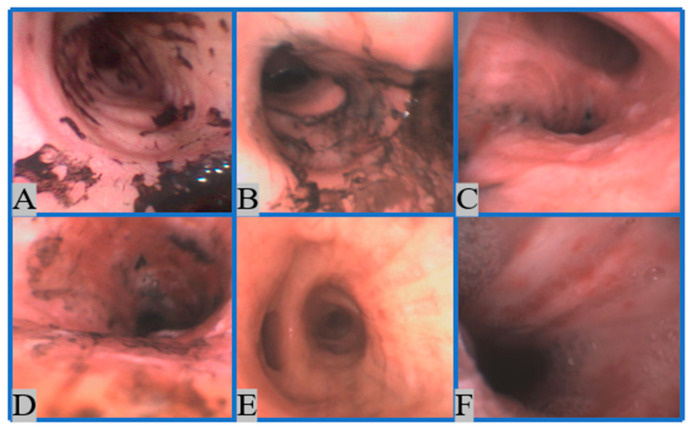
Bronchoscopy images of different degrees based on our proposed grading system. (**A**): degree 1, (**B**): degree 2, (**C**): degree 3, (**D**): degree 4, (**E**): degree 5, (**F**): degree 6.

In this paper, we collect images from eighteen patients’ bronchi and separate them into six groups, corresponding to six categories of inhalation injury severity, according to the period during which patients require mechanical ventilation. The quantity of time requiring mechanical ventilation measured in days is an objective outcome measurement we used to quantify injury severity. The longer the patient required mechanical ventilation, the more severe the injury. We regard (1) extubating under 24 h as degree 1, (2) extubating between 1–2 days as degree 2, (3) extubating between 3–7 days as degree 3, (4) extubating between 8–14 days as degree 4, (5) extubating between 14–30 days as degree 5, and (6) extubating after 30 days as degree 6 as shown in Figure 3. The eighteen patients are divided into (1) two degree 1, (2) six degree 2, (3) three degree 3, (4) two degree 4, (5) three degree 5, and (6) two degree 6.

#### 2.1.2. Image Preprocessing

The initial dataset we collected from the patients has various quality issues, such as blurring and differences in intensity. These various quality issues can cause bias during deep learning model training because of the differences in the image histograms. To minimize the effects of different qualities of the dataset and make the deep learning network focus more on features of inhalation injury, we implement the N-CLAHE algorithm, described by K. Koonsanit et al. [38]. However, before N-CLAHE is used, we convert the initial images into grayscale.

N-CLAHE algorithm consists of two main steps:

1. Normalization

Original images may have some intensities which need to be corrected by adjusting the intensity contrast values by the linear normalization function in (1):

(1) $$I_{N}=\left(I-\mathrm{Min}\right)\frac{\mathrm{newMax}-\mathrm{newMin}}{\mathrm{Max}-\mathrm{Min}}+\mathrm{newMin}$$

where $I_{N}$ is a normalized image, I is an initial image, Max is the maximum intensity value of pixels in the initial image, Min is the minimum intensity value of pixels in the initial image, newMax is the maximum value of the normalized image which we set as 255 in this study, and newMin is the minimum intensity value of the normalized image which we set to 0 in this paper.

2. Contrast-Limited Adaptive Histogram Equalization (CLAHE) [41]

**Figure 4:**
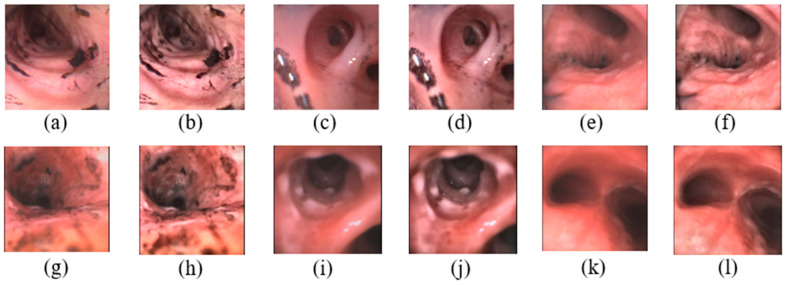
Prepossessed bronchoscopy images from different degrees where (**a**,**c**,**e**,**g**,**i**,**k**) are original images from 1st degree to 6th degree; (**b**,**d**,**f**,**h**,**j**,**l**) are preprocessed images from 1st degree to 6th degree.

The above normalization step enhances an original image in some aspects, e.g., textures and local contrast. The CLAHE process contains three stages: firstly, divide the original image into several nonoverlapping equal-size regions; secondly, calculate the histogram of each region and obtain the clip limit for clipping histograms; thirdly, reassign each histogram so that its height does not exceed the clip limit. The clip limit β can be obtained as in (2):

(2) $$\beta =\frac{M\times N}{L}\left(1+\frac{\alpha }{100}\left(s_{\max}-1\right)\right)$$

where M × N is the number of pixels in each region, L is the number of grayscales, α is a clip factor in (0, 100), and $s_{\max}$ is the maximum allowable slope. In this paper, we set the values of M, N, L, α, and $s_{\max}$ to 8, 8, 256, 40, and −1.4, respectively, which results in a β value of 0.01. When original images are compared with preprocessed images as shown in Figure 4, the intensity variance is reduced, and the details of injuries are enhanced. That will help deep learning models to focus on the features of inhalation injuries.

### 2.2. Method

Machine learning technologies have been widely used in real-world applications. However, labeled training data can often be expensive, inaccessible, or hard to obtain. Transfer learning is proposed to solve this problem by training high-performance models with more easily obtained and larger amounts of data [42]. In this paper, we collected 125 bronchoscopy images from a total of six degrees which are: (1) 13 images of degree 1, (2) 35 images of degree 2, (3) 24 images of degree 3, (4) 22 images of degree 4, (5) 13 images of degree 5, and (6) 18 images of degree 6. The size of our dataset was limited, so transfer learning was applied to solve the size issue and obtain a deep learning model for recognizing the severity of inhalation injury.

#### 2.2.1. Learning and Testing Pipeline

**Figure 5:**
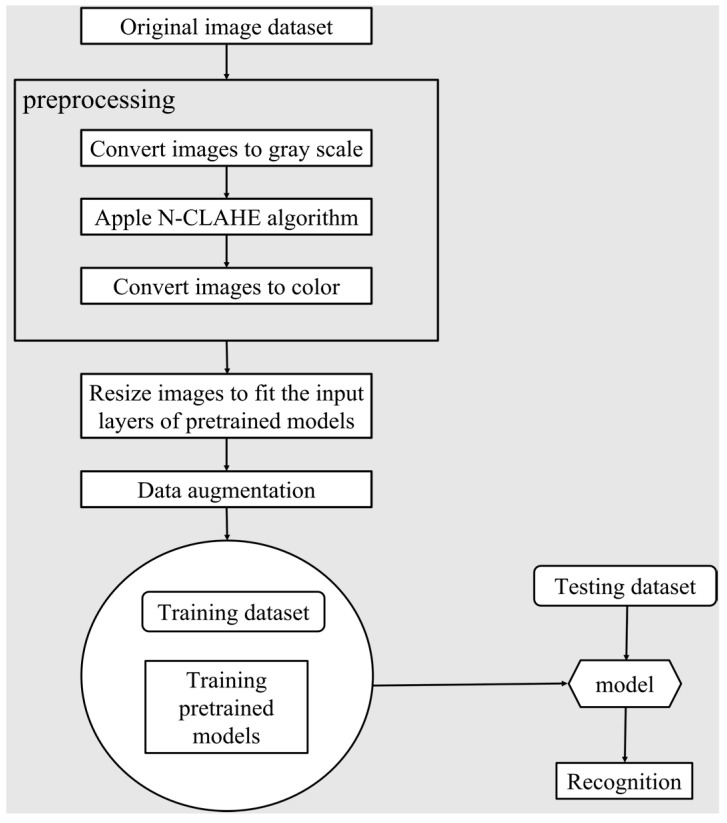
The pipeline of image preprocessing and model development. The original images are converted into gray scales and the N-CLAHE algorithm is applied, after which the images are converted back to color. The images need to be resized to fit the input layers of different models. Data augmentation, including image rotation and scaling, is applied to help fix the issue of limited dataset size. At last, the dataset is divided into the training and testing sets to validate the selected models.

The learning and testing pipeline is shown in Figure 5. The preprocessing is first applied to the original image dataset. In the preprocessing step, the images are converted into grayscale, and their color maps are saved. Then, we applied N-CLAHE algorithm to reduce the intensity variance and enhance the details. After N-CLAHE, these grayscale images are converted back into color with color maps. After preprocessing, the image dataset is resized to fit the input layers of pre-trained models. For example, VGG-16/VGG-19 requires an input image size of 224 × 224 × 3, and SqueezeNet requires an input image size of 227 × 227 × 3.

#### 2.2.2. Data Augmentation

We train deep learning networks with the current dataset, which has a limited size. To avoid overfitting coming from a limited database size, we perform data augmentation after resizing [43]. We confine the way of data augmentation only to the rotation and scaling of our bronchoscopy images to avoid label-changing at some distortion magnitude in data augmentation. The details of the rotation and scaling in our data augmentation are described in the following:

3. Image rotation:

The rotated image output $I_{output}$ is obtained by rotating the original image $I_{input}$ clockwise by positive angles θ as in Equations (3) and (4):

(3) $$x_{output}=cos\theta \left(x_{input}-x_{o}\right)-sin\theta \left(y_{input}-y_{o}\right)+x_{o}$$

(4) $$y_{output}=cos\theta \left(y_{input}-y_{o}\right)+sin\theta \left(x_{input}-x\right)+y_{o}$$

where $\left(x_{output},y_{output}\right)$ are the coordinates of the rotated image, $\left(x_{input},y_{input}\right)$ are the coordinates of the original image, θ is the angle of rotation, and $\left(x_{o},y_{o}\right)$ are the coordinates of the center of rotation.

4. Image scaling:

We implemented the vertical direction scaling, horizontal direction scaling, and the whole-image scaling models. Bilinear interpolation is applied in image scaling. When we just scale the image in one direction, horizontally (*x*) or vertically (*y*), we apply Equations (5) and (6), of which there is an example in the *x* direction:

(5) $$Pixel\left(x,y_{1}\right)=\frac{x_{2}-x}{x_{2}-x_{1}}Pixel\left(x_{1},y_{1}\right)+\frac{x-x_{1}}{x_{2}-x_{1}}Pixel\left(x_{2},y_{1}\right)$$

(6) $$Pixel\left(x,y_{2}\right)=\frac{x_{2}-x}{x_{2}-x_{1}}Pixel\left(x_{1},y_{2}\right)+\frac{x-x_{1}}{x_{2}-x_{1}}Pixel\left(x_{2},y_{2}\right)$$

where $Pixel\left(x_{i},y_{i}\right)$ is the pixel value in the position $\left(x_{i},y_{i}\right)$ of the original image, *x* is the coordinate in the horizontal direction of the interpolated pixel, ($x_{1},y_{1}$), ($x_{2},y_{1}$), ($x_{1},y_{2}$), ($x_{2},y_{2}$) are the four nearest points in the original image, with $x_{2}=x_{1}+1$, $y_{2}=y_{1}+1$.

When applying the whole-image scaling, we followed Equation (7):

(7) $$\begin{array}{c} Pixel\left(x,y\right)\\ =\frac{1}{\left(x_{2}-x_{1}\right)\left(y_{2}-y_{1}\right)}\left[\begin{array}{cc} x_{2}-x & x-x_{1} \end{array}\right]\left[\begin{array}{cc} Pixel\left(x_{1},y_{1}\right) & Pixel\left(x_{1},y_{2}\right)\\ Pixel\left(x_{2},y_{2}\right) & Pixel\left(x_{2},y_{2}\right) \end{array}\right]\left[\begin{array}{c} y_{2}-y\\ y-y_{1} \end{array}\right] \end{array}$$

where *y* is the coordinate in the vertical direction of the interpolated pixel.

Image reflection is not applied in either horizontal or vertical directions as data augmentation in this paper. Because bronchoscopy images are not symmetrical (central, vertical, or horizontal), the reflection may cause distortion. Data augmentation extends the size of the dataset from 125 images to 1000 images. We divide these images into 70% training and 30% testing sets. The training set is used to train the selected models, and the testing set is used to test trained models with performance metrics, including precision, sensitivity, specificity, accuracy, and F1 score.

#### 2.2.3. Transfer Learning

**Figure 6:**
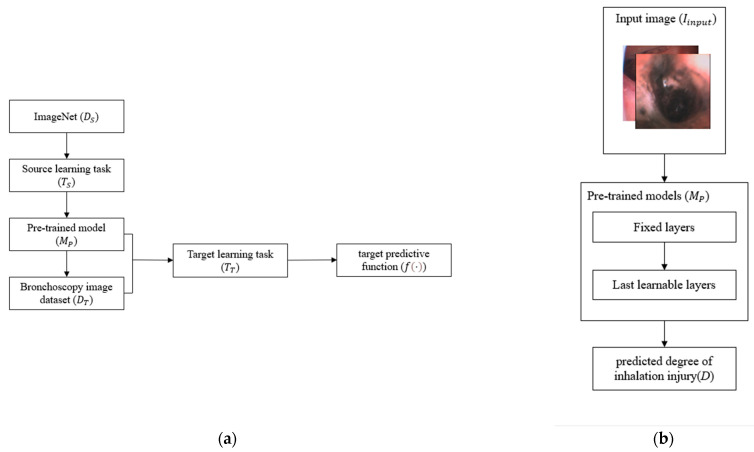
(**a**) The flow chart of training transfer measure the target predictive function f(·) from $D_{T}$ in $T_{T}$. (**b**) The diagram of how the layers in pre-trained models ($M_{P}$) help to predict the degree of inhalation injury (*D*).

Transfer learning is an approach that enhances the performance of traditional machine learning methods when the database size is small [42]. We consider the ImageNet database, which contains 1000 classes and 14,000,000 labeled images, as the source domain $D_{S}$, with an average image resolution of 496 × 387 × 3. The source learning task $T_{S}$ is the training process that was used to train and obtain the pre-trained model $M_{P}$. At the same time, the bronchoscopy image dataset developed in this paper was regarded as the target domain $D_{T}$, with an average image resolution of 512 × 512. The target learning task $T_{T}$ is the process used to figure out how to grade the severity of inhalation injuries, where $D_{S}\neq D_{T}$, and $T_{S}\neq T_{T}$. In this case, transfer learning contributed to measuring the target predictive function f(·) from $D_{T}$ in $T_{T}$ as shown in Figure 6a, which determined the injury degree from bronchoscopy result [44] in Equation (8):

(8) $$D=f\left(I_{input}\right)$$

where *D* is the predicted degree of inhalation injury, $I_{input}$ is the input image fed in the model.

The flow chart of transfer learning training is shown in Figure 6b; transfer learning is a “black box” method, which means that we can use the pre-trained models directly to simplify the training process. For example, if VGG-16 [29] is selected as our pre-trained model, it will be divided into two parts: fixed layers and learnable layers. We keep all the weights in fixed layers, which consist of convolutional layers and ReLU layers, to simplify the training because these layers take information from the previously learned task with ImageNet. Indeed, they have the potential to recognize the characteristics in images. VGG-16 can recognize 1000 classes; however, there are six grades of inhalational injuries. Hence, adjusting the size of the fully connected layer and SoftMax layer from 1 × 1 × 1000 to 1 × 1 × 6 and retraining these layers are necessary; these layers become the last learnable layers. Then, the retrained VGG-16 model will recognize the grades of input images.

#### 2.2.4. Model Selection

Deep convolutional neural network (CNN) models are outstanding at analyzing images. Transfer learning based on deep CNN has proven effective in medical image classification [45]. In this study, we select six pre-trained deep CNN-based models and retrain the weights in the last learnable layers, which are used to predict the probability of the image belonging to each category, using the training set. Then, the testing set was used to evaluate the performance of the updated models. To find the optimized model, we focused on which models are widely used in transfer learning and available in packaged form through trusted public libraries, such as Keras. This is discussed below.

1. VGG-16

VGG-16 is a CNN-based model used in object detection and the classification of up to 1000 different categories with high accuracy. Its architecture has small convolution filters, which make the network converge faster. They also reduce the number of weight parameters so that the tendency to overfit decreases. The fully connected and SoftMax layers were retrained to fit our six categories.

2. VGG-19

VGG-19 [29] is also a CNN-based model with a similar architecture to VGG-16, both of which use 3 × 3 filters to improve accuracy. The difference between VGG-16 and VGG-19 is that VGG-16 has 16 layers in the base model, while VGG-19 has 19 layers. The fully connected and SoftMax layers were retrained to six dimensions, thus fitting the six categories.

3. SqueezeNet

SqueezeNet [46] is a CNN-based model with smaller architecture. The base model was pre-trained on more than a million images from the ImageNet dataset and provided high computational efficiency and achieved AlexNet-level [30] accuracy on ImageNet with fewer parameters. In this paper, we trained the convolution layer at the end of the model and the SoftMax layer to fit six degrees.

4. ResNet-18

ResNet-18 [33] is a CNN-based model which is 18 layers deep. A residual learning framework is present to ease the training of deeper networks. This network has lower complexity with deeper architecture, preventing overfitting. The fully connected layer and the SoftMax layer were trained to recognize six categories.

5. ResNet-50

ResNet-50 [33] is a CNN-based model which is 50 layers deep. Like ResNet-18, more complex residual blocks are also used in ResNet-50 to prevent overfitting and the gradient from vanishing. The fully connected layer and the SoftMax layer were still trained for six categories in this study.

6. GoogLeNet

GoogLeNet [47] is a CNN-based model which is 22 layers deep. It uses inception architecture to reduce the number of parameters, including weights and biases. Global average pooling is applied at the end of the network instead of a fully connected layer, decreasing the number of trainable parameters. The averaging layer and the SoftMax layer at the end of the network were also trained to fit our six categories.

7. CNN-13

**Figure 7:**
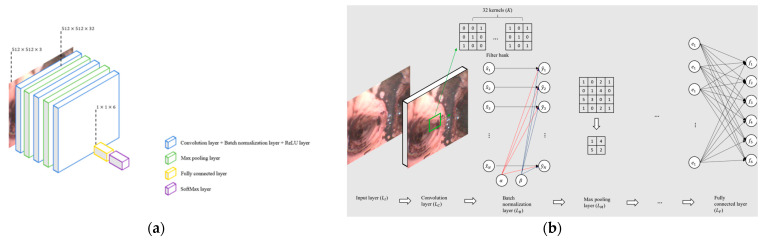
(**a**) Architecture of CNN-13, which is a typical 13 layers deep CNN model (includes 3 convolution layers). (**b**) The details of how hidden layers in CNN-13 work.

We designed two typical CNN models to compare transfer learning and traditional image classifiers: CNN-13 and CNN-25. CNN-13 is 13 layers deep: the 3 convolutional layers, 2 max-pooling layers, and the fully connected layer can learn the features of the image dataset, and the SoftMax layer provides the probability of the image belonging to the corresponding grade. The architecture of CNN-13 is shown in Figure 7a.

In convolution layers ($L_{C}$), we applied 3 × 3 kernel (*K*) and stride (*s*) as 1. A total of 32 kernels formed the filter bank where the kernels were connected to the same region for collecting features such as the green square, shown in Figure 7b of the output of the previous layer. Batch normalization is a widely used regularization technique which is normally activated by Equation (9):

(9) $${\hat{x}}_{i}=\frac{x_{i}-\mu_{B}}{\sqrt{\sigma_{B}{}^{2}}}$$

where $x_{i}$ is the input elements of the batch normalization layer ($L_{B}$), which is composed also of output elements from $L_{C}$. $\mu_{B}$ and $\sigma_{B}$ are the mean and variance over the dimensions (*d*) of input. Further activation was applied following Equation (10):

(10) $${\hat{y}}_{i}=\gamma +\delta {\hat{x}}_{i}$$

where *δ* and *γ* are learnable parameters updating during training.

Then, the ReLU function was used as the nonlinear activation function after $L_{B}$, as shown in Equation (11):

(11) $$g\left(x\right)=\{\begin{array}{c} x\;\;\;\;\;x\geq 0\\ 0\;\;\;\;\;x<0 \end{array}$$

where *x* represents each element ${\hat{y}}_{1}$–${\hat{y}}_{N}$ from the output matrix $Y_{B}$ of batch normalization layers, as shown in Figure 7b, and N is the number of elements in $Y_{B}$. ReLU function thresholds represent each input element of the ReLU layer where any value less than zero will be set to zero.

SoftMax function in Equation (12) was used to measure the probability, $P_{j}$, of the input image belonging to the corresponding degree *j* from the output $f_{j}$ of the fully connected layer:

(12) $$P_{j}\left(f\right)=\frac{e^{f_{j}}}{\sum_{i=1}^{N_{I}}e^{f_{i}}}$$

where j∈{1,2,3,4,5,6} is the serial number of injury degree, $f_{j}$ is the output from a fully connected layer of degree *j*, $N_{I}$ is 6 in this case which means there are 6 degrees, $f_{i}$ is output from the fully connected layer with the index number *i*, and $P_{j}\left(x\right)$ is the probability of the input image belonging to degree *j.*

8. CNN-25

**Figure 8:**
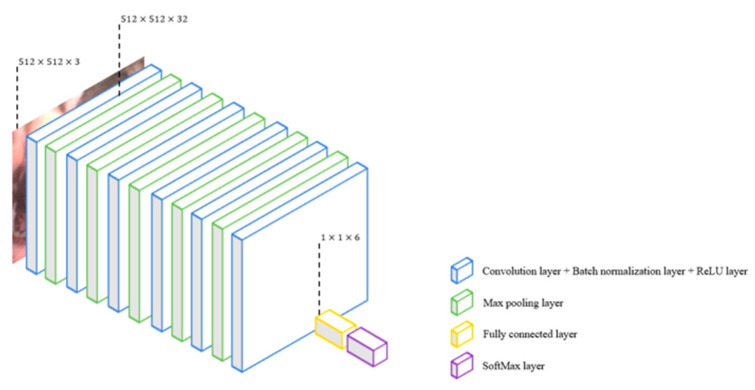
Architecture of CNN-25, which is a typical 25-deep CNN model (includes 6 convolution layers).

Similar to CNN-13, CNN-25 is also a typical CNN-based model which is 25 layers deep. We inserted six more convolution layers to observe if the accuracy of a typical CNN-based model increases with a deeper architecture. The architecture of CNN-25 is shown in Figure 8.

#### 2.2.5. Experiment Set Up

In this study, we finished all experiments with Matlab 2022a and the deep network designer developed by MathWorks. Individual experiments are designed for each base model to achieve the best performance metrics. Multiple hyperparameter ranges are tested to fit different models, as shown in Table 1. A larger learning rate makes the model learn faster while obtaining suboptimal weights; a smaller learning rate helps the model to achieve more optimal weights while taking a longer time to train. In this study, we apply a learning rate schedule, dropping the learning rate during training instead of relying on a fixed learning rate to optimize the learning rate [48]. The initial learning rate is updated every period, which is a certain number of epochs, by multiplying with a certain factor. Mini-batch [49], a typical method to smoothen the gradient descent during training, is also applied in this study and shuffled every epoch. We also performed comparison experiments without data augmentation to analyze the impact of data augmentation.

## 3. Results

**Figure 9:**
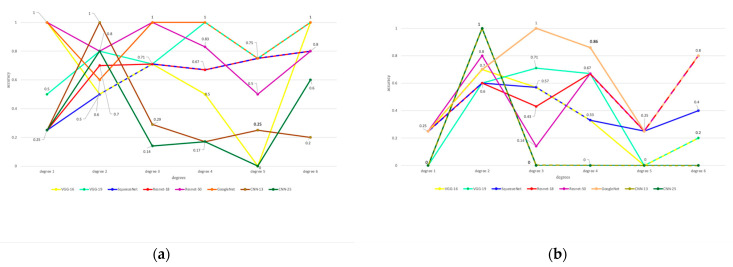

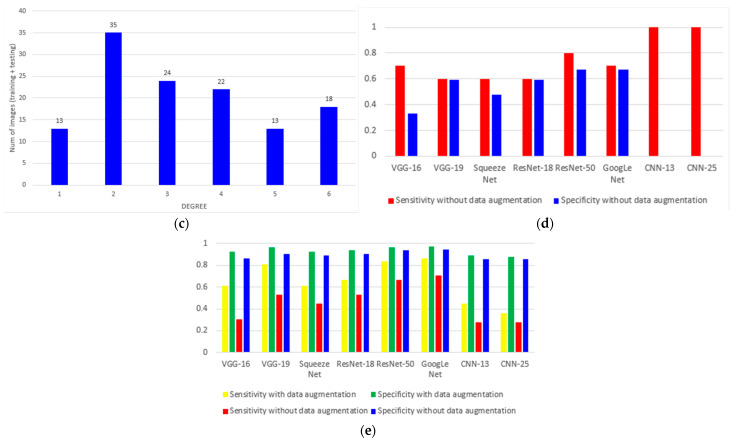
The accuracy in each degree with different models, where red dots represent the accuracy of VGG-16, green dots represent the accuracy of VGG-19, blue dots represent the accuracy of SqueezeNet, yellow dots represent the accuracy of ResNet-18, purple dots represent the accuracy of ResNet-50, orange dots represent the accuracy of GoogLeNet, rosy-brown dots represent the accuracy of CNN-13, and khaki dots represent the accuracy of CNN-25. (**a**) Accuracy over each degree with augmentation. (**b**) Accuracy over each degree without augmentation. (**c**) The dataset distribution contains both the testing set and the training set. (**d**) Comparison of sensitivity and specificity over each method with and without data augmentation on degree 2 only. (**e**) Comparison of average sensitivity and specificity over each method with and without data augmentation over all degrees.

The accuracy in each degree with different models is shown in Figure 9, where red, green, blue, yellow, purple, orange, rosy-brown, and khaki dots represent the accuracies of VGG-16, VGG-19, SqueezeNet, ResNet-18, ResNet-50, GoogLeNet, CNN-13, and CNN-25, and the dotted lines represent the overlapped results, respectively. Specifically, Figure 9a,b shows the accuracies with and without data augmentation, respectively. As shown in Figure 8, data augmentation improves by 25–75% in degree 1, 10–20% in degree 2, 14–86% in degree 3, 14–34% in degree 4, 25–75% in degree 5, and 20–80% in degree 6. Besides, the models trained without data augmentation can provide 20%, 10%, 10%, and 20% higher accuracies in degree 2 with VGG-16, SqueezeNet, GoogLeNet, and CNN-25.

We also use performance metrics which consist of (1) precision, (2) sensitivity, (3) specificity, (4) accuracy, and (5) F-measure (F1-score) to evaluate optimized models; these indexes are calculated with Equations (13)–(17) for every individual degree. In our multi-class case, performance metrics are calculated separately, degree by degree, with the one versus all method being used. This regards one degree as degree A and the other degrees as degree B [50]. For example, when we calculate performance metrics of degree 2, degree 2 is regarded as degree A, and the other degrees (1, 3, 4, 5, 6) are regarded as degree B, where true positive (TP) counts the number of images that the model recognized in degree A correctly; false positive (FP) counts the number of images that the model misrecognized from degree B as in degree A; true negative (TN) counts the number of images that the model recognized correctly as being in degree B; false negative (FN) counts the number of images that the model misrecognized from degree A as in degree B. Specifically, the sensitivity, in this case, refers to the ability of the network identify each degree. Additionally, the specificity refers to the ability of the network to correctly recognize the images which do not belong to each degree [50].

(13) $$\mathrm{precision}=\frac{\mathrm{TP}}{\mathrm{TP}+\mathrm{FP}}$$

(14) $$\mathrm{sensitivity}=\frac{\mathrm{TP}}{\mathrm{TP}+\mathrm{FN}}$$

(15) $$\mathrm{specificity}=\frac{\mathrm{TN}}{\mathrm{TN}+\mathrm{FP}}$$

(16) $$\mathrm{accuracy}=\frac{\mathrm{TP}+\mathrm{TN}}{P+N}$$

(17) $$F1\;\mathrm{score}=2\frac{\mathrm{precision}\cdot \mathrm{sensitivity}}{\mathrm{precision}+\mathrm{sensitivity}}$$

Our current dataset is not balanced, and the dataset distribution is shown in Figure 9c, where the images in degrees 1, 2, 3, 4, 5, and 6 take 10.4%, 28%, 19.2%, 17.6%, 10.4%, and 14.4%, respectively. In Figure 9d, we compare the specificity and sensitivity of each method on degree 2 without data augmentation, where the networks provide higher sensitivity and lower specificity at the same time. This suggests that the false positive predictions are abnormal high, and the networks tend to predict input images as degree 2 no matter the images belong to.

In Figure 9e, the average values of sensitivity and specificity over all degrees from each model are shown, and it can be seen that the models trained with data augmentation provide higher specificity and sensitivity. This indicates that data augmentation reduces the false negative and false positive rates, which means fewer cases of injury are missed and fewer cases are classified to other degrees.

Besides transfer learning models, we designed and tested two typical CNN-based models: CNN-13 and CNN-25. As shown in Figure 9e, the sensitivity and specificity of CNNs are always lower than those of transfer learning methods with and without data augmentation.

The micro average performance metric values of six degrees are used to represent the performance of the methods. The performance metrics of the optimized models are shown in Table 2 where the bold in the table highlights best performance over all methods, and GoogLeNet achieves the best performance with the highest index values. It is also observed that CNN-25 and CNN-13 could not achieve the accuracy that transfer learning reached.

We also compare the performance metrics of each model, both with and without data augmentation. The performance metrics of optimized models without data augmentation are shown in Table 3 where the bold in the table highlights best performance over all methods. Comparing the corresponding values in Table 2 and Table 3, we can observe data augmentation obviously increases the performance of deep models and the accuracy of the severity grading.

Additionally, to determine if the depth of models increasing causes overfitting in our case, we compared VGG-16/VGG-19, CNN-13/CNN-25, and ResNet-18/ResNet-50. It is observed that the accuracy of VGG-19 improves by 19.45% and 22.22% with and without data augmentation compared to VGG-16, and the accuracy of ResNet-50 improves by 16.66% and 13.89% with and without data augmentation compared to ResNet-18. However, the accuracy of CNN-25 decreases by 8.33% with data augmentation compared to CNN-13.

## 4. Discussion

Table 2 shows that the typical CNN-based model’s accuracy is between 36.11% and 44.44%, which is always lower than transfer learning. The accuracy even declines when the depth of the model increases since the limited size of our current dataset causes overfitting when the complexity of the model grows. In contrast, the overfitting issue does not happen to the transfer learning methods. For example, when we compare the following pairs of transfer learning methods, (1) VGG-16 versus VGG-19, (2) ResNet-18 versus ResNet-50, the model’s accuracy increases as the depth grows. This proportional relation between the accuracy and the depth may arise because the pre-trained weights and biases in fixed layers, introduced in Section 2.2.2, may effectively reduce the complexity of models during training, which results in only 2–3 learnable layers needing to be updated.

Figure 9c shows the total number of images used for training or testing the methods. In this paper, grade 2 images are relatively more common (28% of total images) than the other grades 1, 3, 4, 5, and 6. This higher percentage of grade 2 images leads to a higher a priori probability in prediction, which causes these networks to tend to classify the input images to degree 2, no matter what degree the images belong to.

Due to this data imbalance, performance metrics such as sensitivity and specificity may be biased if the data augmentation or transfer learning is not included. Table 2 and Table 3 show data augmentation’s impact on all the performance metrics: accuracy, sensitivity, specificity, F-1 score, and precision. Conversely, Figure 9e only visualizes each method’s average sensitivity and specificity with and without data augmentation among these performance metrics. As shown in Figure 9e, the average sensitivity and specificity increase when data augmentation is applied since the expanded size of the dataset coming from the data augmentation reduces overfitting during training. Moreover, transfer learning employed in VGG-16, VGG-19, SqueezeNet, ResNet-18, ResNet-50, and GoogLeNet gives higher sensitivity and specificity than CNN-13 and CNN-25, which do not have the transfer learning component. Furthermore, more hidden layers improve the sensitivity and specificity of the methods using the transfer learning component, while the more hidden layers did not improve the sensitivity and specificity of the method which does not have the transfer learning component, as shown in Figure 9e.

To analyze only the impact of the transfer learning, we did not include the data augmentation component for each method in Figure 9d. As shown in Figure 9d, the specificity values of CNN-13 and CNN-25 without data augmentation are zero, while the specificity values of the other methods are not. This is because CNN-13 and CNN-25 are not equipped with the transfer learning component, so the ability to correctly classify non-degree 2 data into non-degree 2 is low, i.e., the specificity value is small when the data augmentation is not implemented. Hence, the specificity value is seen to be vulnerable to the lack of both transfer learning applications. Moreover, Figure 9d shows that CNN-13 and CNN-25 give 100% sensitivity, while they give 0% specificity values without data augmentation in terms of grade 2. This indicates that the unbalanced dataset distribution led to networks having a higher probability of predicting the degree which takes the highest percentages in the dataset.

The novelty of this study and method cannot be understated. Currently, there is no objective way to diagnose and grade an inhalation injury. This is entirely dependent on the healthcare provider, who makes a subjective determination. This study aims to establish an objective and consistent way for providers to diagnose and grade inhalation injuries. Additionally, establishing a standardized and objective method to diagnose inhalation injuries will allow providers to care for their patients more accurately by understanding the full and accurate extent of their inhalation injury. This will be especially important given that inhalation injuries are complications historically associated with higher mortality and morbidity in the burn population.

In conclusion, our experimental results show that our proposed transfer learning method performs with higher accuracy than the typical CNN-based model, which is a non-transfer learning method, and that data augmentation improves the average accuracy. Specifically, the proposed algorithm with GoogLeNet provides the highest average accuracy of 86.11%. It indicates that grading the severity of inhalation injury can be predicted by bronchoscopy images. According to the expected grading of the severity, the period of mechanical ventilation will be determined by clinicians. Since the dataset is limited in this paper, evaluating and improving our proposed algorithm with a larger dataset will be required to use this approach to aiding burn surgeons in clinics. Hence, our future work will involve collecting a larger amount of bronchoscopy images from patients who suffer inhalation injuries and training the model with these images. In this procedure, more understanding of a larger dataset is expected to improve our proposed method. As a result, the improved method is expected to make the first step to standardizing therapy in an efficacious manner by overcoming the current limitations of bronchoscopy-based severity estimation of inhalational injuries, such as a lack of accuracy and consistency in each degree and an unbalanced dataset. This is will be achieved by objectifying the process of inhalational injury grading, and developing an assisting tool determining the period of mechanical ventilation based on objectively predicted injury grades.

## Figures and Tables

**Table 1:** Experiment setting.

Hyperparameters	Range
Initial learning rate (l)	$10^{-5}$–$10^{-3}$
Learning rate drop period (LP)	5–15
Learning rate drop factor (LF)	0.05–0.2
Max epochs (ME)	10–50
Mini-batch size (MB)	2–6

**Table 2:** Performance metrics of retrained models and typical CNN models with data augmentation.

Model	Precision	Sensitivity	Specificity	Accuracy	F1 Score
VGG-16	61.11%	61.11%	92.22%	61.11%	61.11%
VGG-19	80.56%	80.56%	96.11%	80.56%	80.56%
Squeeze Net	61.11%	61.11%	92.22%	61.11%	44.44%
ResNet-18	66.67%	66.67%	93.33%	66.67%	66.67%
ResNet-50	83.33%	83.33%	96.67%	83.33%	83.33%
GoogLeNet	**86.11%**	**86.11%**	**97.22%**	**86.11%**	**86.11%**
CNN-13	44.44%	44.44%	88.89%	44.44%	44.44%
CNN-25	36.11%	36.11%	87.22%	36.11%	36.11%

**Table 3:** Performance metrics of retrained models and typical CNN models without data augmentation.

Model	Precision	Sensitivity	Specificity	Accuracy	F1 Score
VGG-16	30.56%	30.56%	86.11%	30.56%	30.56%
VGG-19	52.78%	52.78%	90.56%	52.78%	52.78%
Squeeze Net	44.44%	44.44%	88.89%	44.44%	44.44%
ResNet-18	52.78%	52.78%	90.56%	52.78%	52.78%
ResNet-50	66.67%	66.67%	93.33%	66.67%	66.67%
GoogLeNet	**70.27%**	**70.27%**	**94.05%**	**70.27%**	**70.27%**
CNN-13	27.78%	27.78%	85.56%	27.78%	27.78%
CNN-25	27.78%	27.78%	85.56%	27.78%	27.78%
